# Supplementary material for: Characterization of extracellular vesicles and synthetic nanoparticles with four orthogonal single‐particle analysis platforms
Source: J Extracell Vesicles. 2021 Apr 6;10(6):e12079. doi: 10.1002/jev2.12079 (PMC8023330; doi:10.1002/jev2.12079)
Supplement: Supplementary file 1 — Supporting information. [file JEV2-10-e12079-s008.docx]

**Supplementary Table 1. TEM and DLS measurements of selected PS beads**. Three preparations of each PS bead population were measured three times each. Avg = arithmetic mean; SD = standard deviation

| **Nominal diameter (nm)** | **Diameter per data sheet (nm)** | **TEM: Avg diameter (nm) +/- SD (nm)** | **DLS: Z-avg diameter (nm) +/- SD (nm)** | **DLS: Polydispersity index +/- SD** |
| --- | --- | --- | --- | --- |
| 70 | 70 +/- 3 | 70.5 +/- 4.8 | 72.8 +/- 0.8 | 0.03 +/- 0.01 |
| 90 | 92 +/- 3 | 91.1 +/- 5.3 | 91.0 +/- 0.6 | 0.07 +/- 0.01 |
| 125 | 125 +/- 3 | 119.0 +/- 6**.**1 | 114.6 +/- 0.3 | 0.01 +/- 0.00 |
| 150 | 147 +/- 3 | 147.7 +/- 5.2 | 129.5 +/- 6.6 | 0.16 +/- 0.03 |

**Supplementary Table 2: Antibodies tested with fluorescent NTA.** H9 100K EVs were diluted 1:1 (v:v) in PBS. 9 µL of diluted EVs were mixed with 1 µL of antibody and incubated for 2 hours at room temperature. Samples were then diluted 1:1000 and measured in scatter and fluorescent modes using NTA. We would like to stress that our inability to obtain signal with these antibodies likely indicates that further optimization is needed, not necessarily that the antibodies are unsuited to this use.

| **Tetraspanin** | **Fluorophore** | **Manufacturer** | **Catalog Number** | **Signal** |
| --- | --- | --- | --- | --- |
| CD81 | AF488 | Santa Cruz | sc-166029 | No |
|  | PE | BD Biosciences | BDB555676 | Yes |
|  | PerCP | BD Biosciences | BDB565430 | No |
|  | APC | BD Biosciences | BDB561958 | No |
| CD63 | AF488 | Santa Cruz | sc-5275 | No |
|  | AF488 | Novus Biologicals | NBP2-42225 | No |
|  | PE | AbCam | ab205540 | No |
|  | V450 | BD Biosciences | BDB561984 | No |
| CD9 | PE | BioLegend | 312106 | No |
|  | PerCP | BD Biosciences | BDB561329 | No |
|  | FITC | AbCam | ab34162 | No |
