# Supplementary figures and images for: Characterization of extracellular vesicles and synthetic nanoparticles with four orthogonal single‐particle analysis platforms
Source: J Extracell Vesicles. 2021 Apr 6;10(6):e12079. doi: 10.1002/jev2.12079 (PMC8023330; doi:10.1002/jev2.12079)

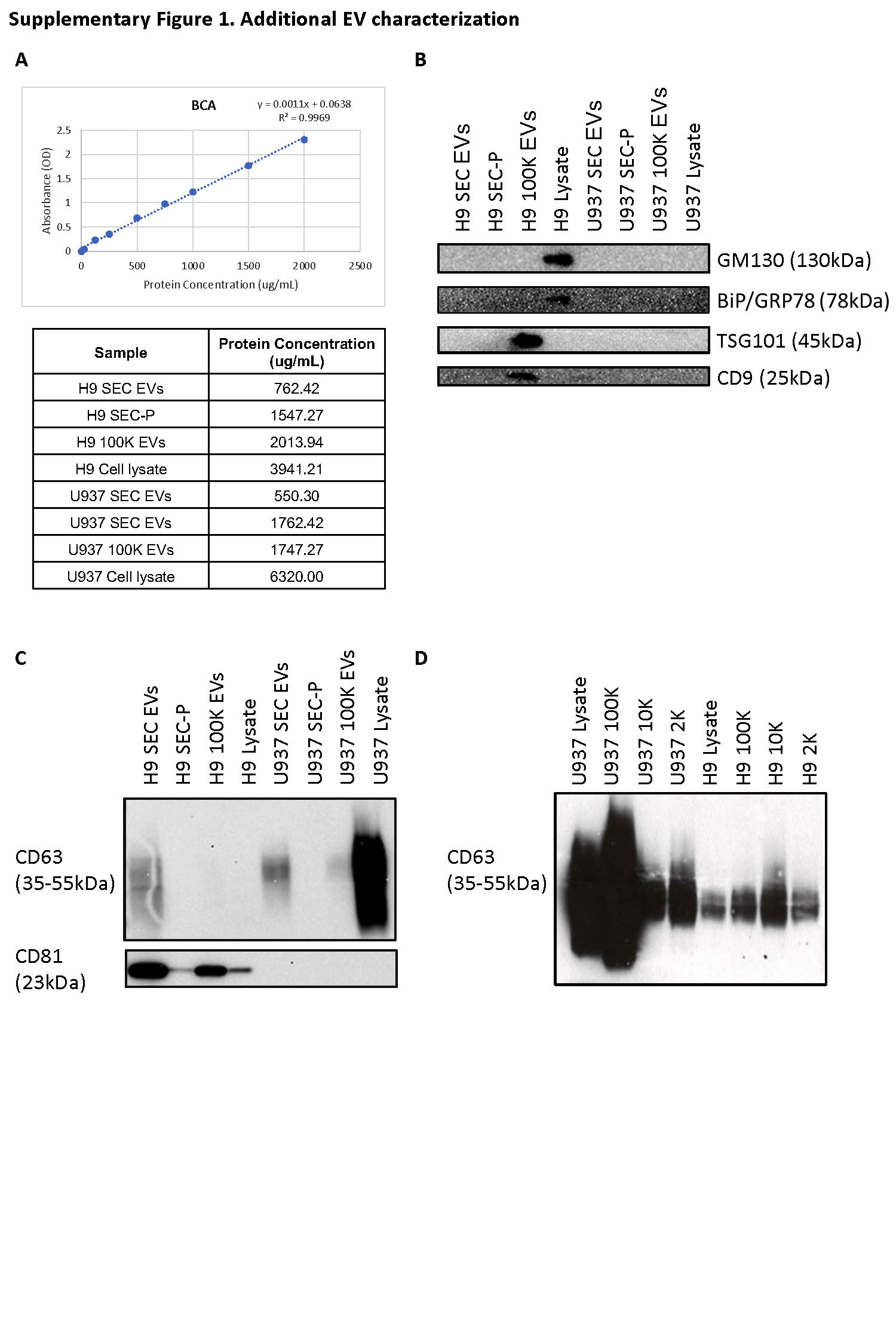

Supplement: Supplementary file 2 — Supplementary Figure 1: Additional EV characterization. (A) Representative BCA assay (protein concentration) results from one batch of EV separations. (B) Immunoblot analysis of separated EVs using the same samples as in Figure 1 but probing for GM130 and BiP/GRP78 (expected to be depleted in EVs) and TSG101 and CD9 (expected to be enriched in EVs). (C) An immunoblot from a previous experiment that was shown in Figure 1 of a previous version of this manuscript. (D) Overexposed CD63 results from a previous set of EV separations from U937 and H9 cells (here, using differential ultracentrifugation at 2K × g, 10K × g, and 100K × g) showing that, in some experiments, CD63 is indeed enriched in the 100K EV‐enriched pellet, and that lengthy exposure confirms the presence of CD63 in H9 cell lysate, albeit at low levels. [file JEV2-10-e12079-s004.jpg]

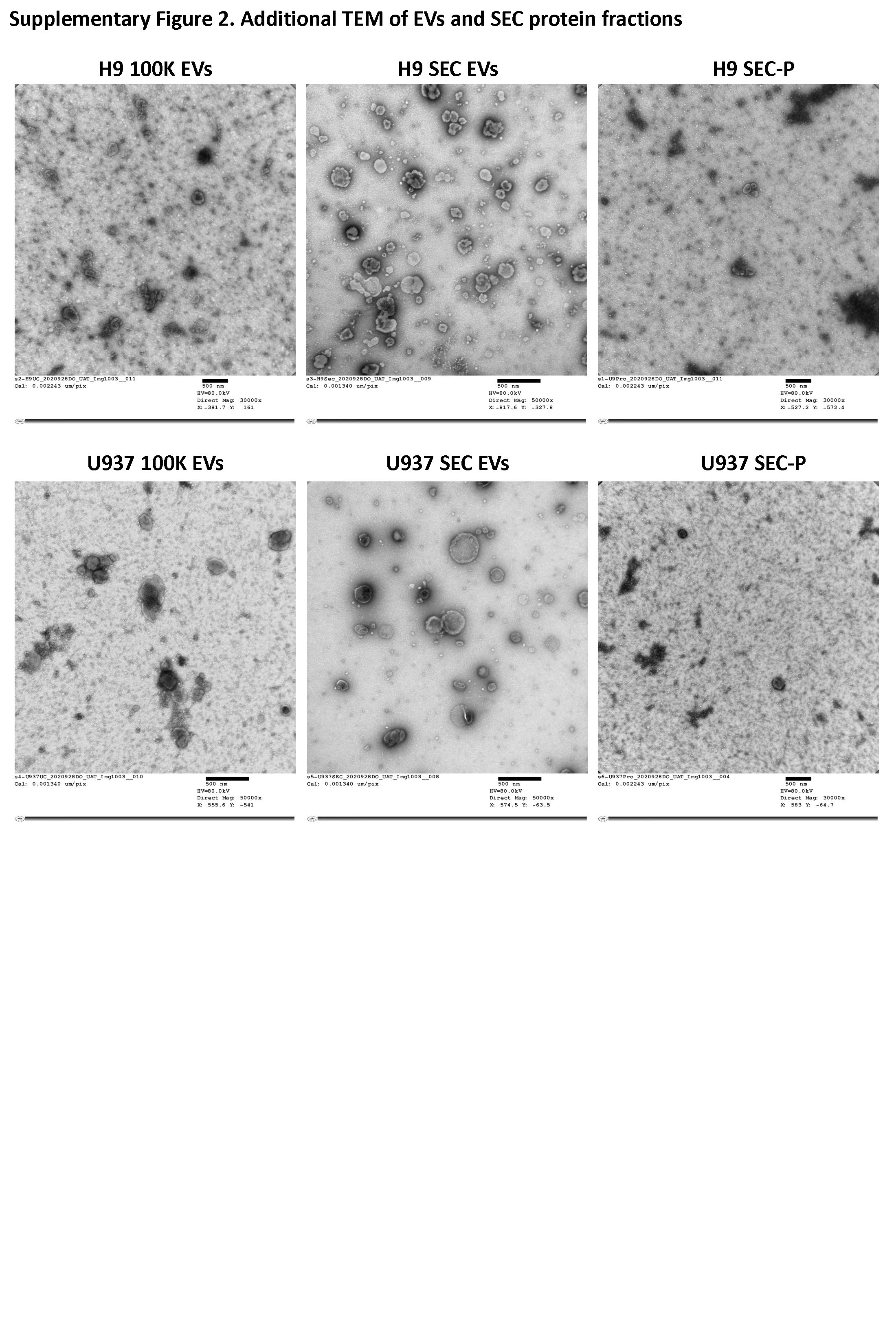

Supplement: Supplementary file 3 — Supplementary Figure 2: Additional TEM of EVs and SEC protein fractions. Scale bars, as indicated, are 500 nm. [file JEV2-10-e12079-s007.jpg]

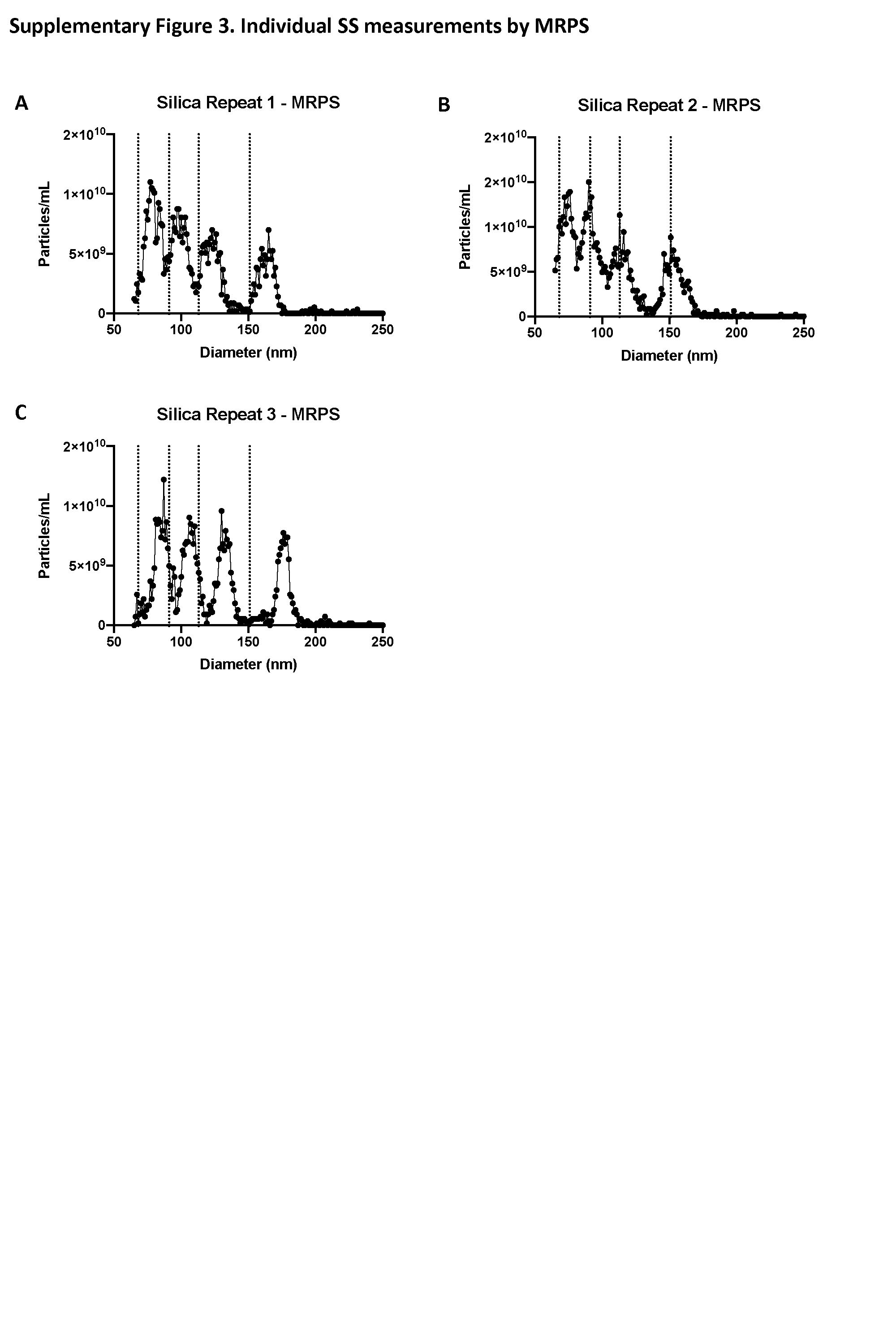

Supplement: Supplementary file 4 — Supplementary Figure 3: Individual SS measurements by MRPS. Repeat 1 (A) can also be found as an inset in Figure 2C. (B) and (C) are additional repeats using the same SS mixture. [file JEV2-10-e12079-s001.jpg]

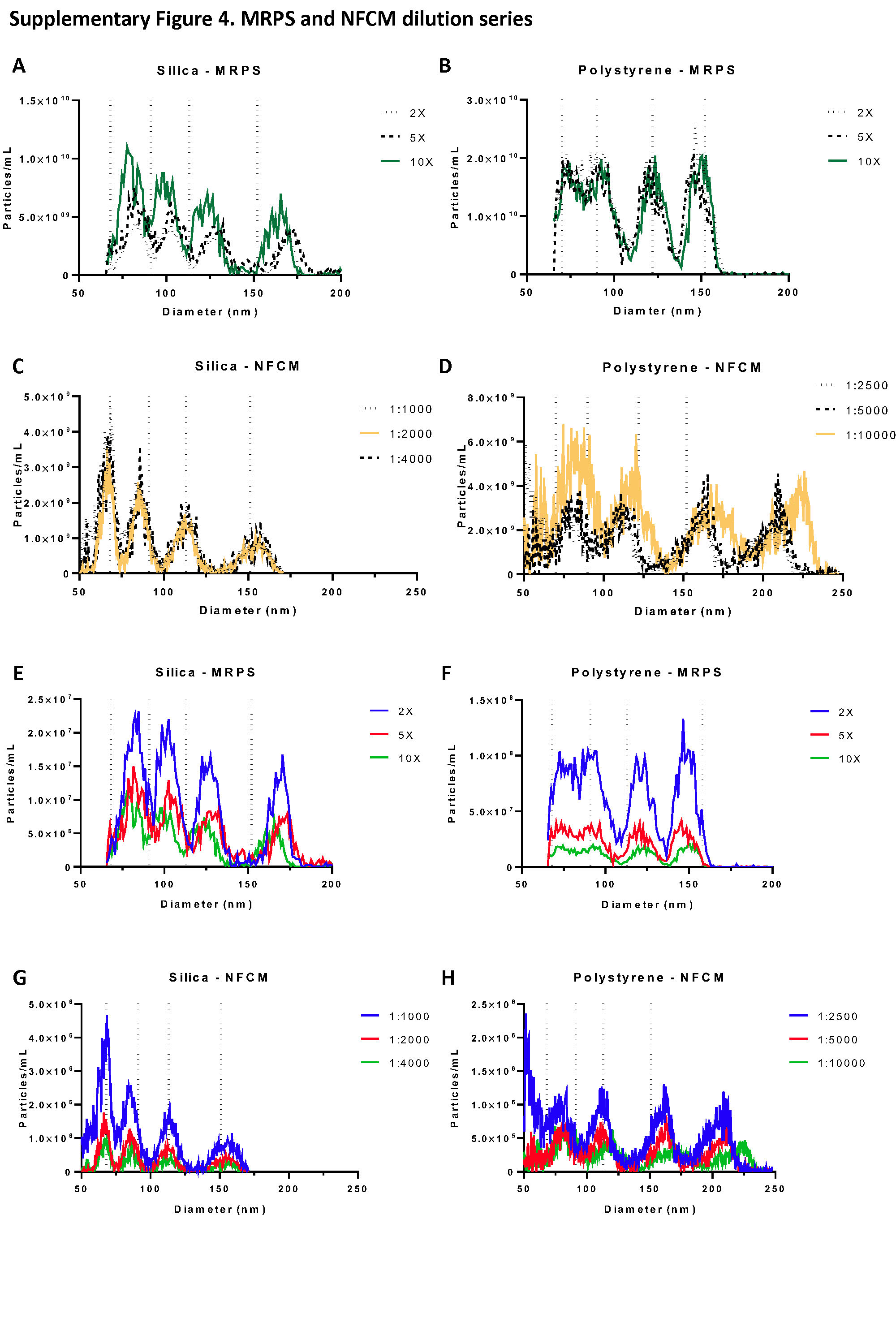

Supplement: Supplementary file 5 — Supplementary Figure 4: MRPS and NFCM dilution series. SS (A) and PS (B) were diluted 2×, 5× and 10× by volume to determine the optimal dilution for NTA analysis. SS (C) were diluted 1:1000 (v:v), 1:2000 (v:v), and 1:4000 (v:v) and PS (D) were diluted 1:2500 (v:v), 1:5000 (v:v) and 1:10000 (v:v) to determine the optimal dilution for NFCM analysis. The upper four panels are dilution‐corrected data. Bottom four panels: raw data for SS (E) and PS (F) on MRPS and SS (G) and PS (H) on NFCM. Optimal dilutions are indicated by green or yellow (MRPS and NFCM, respectively). [file JEV2-10-e12079-s002.jpg]

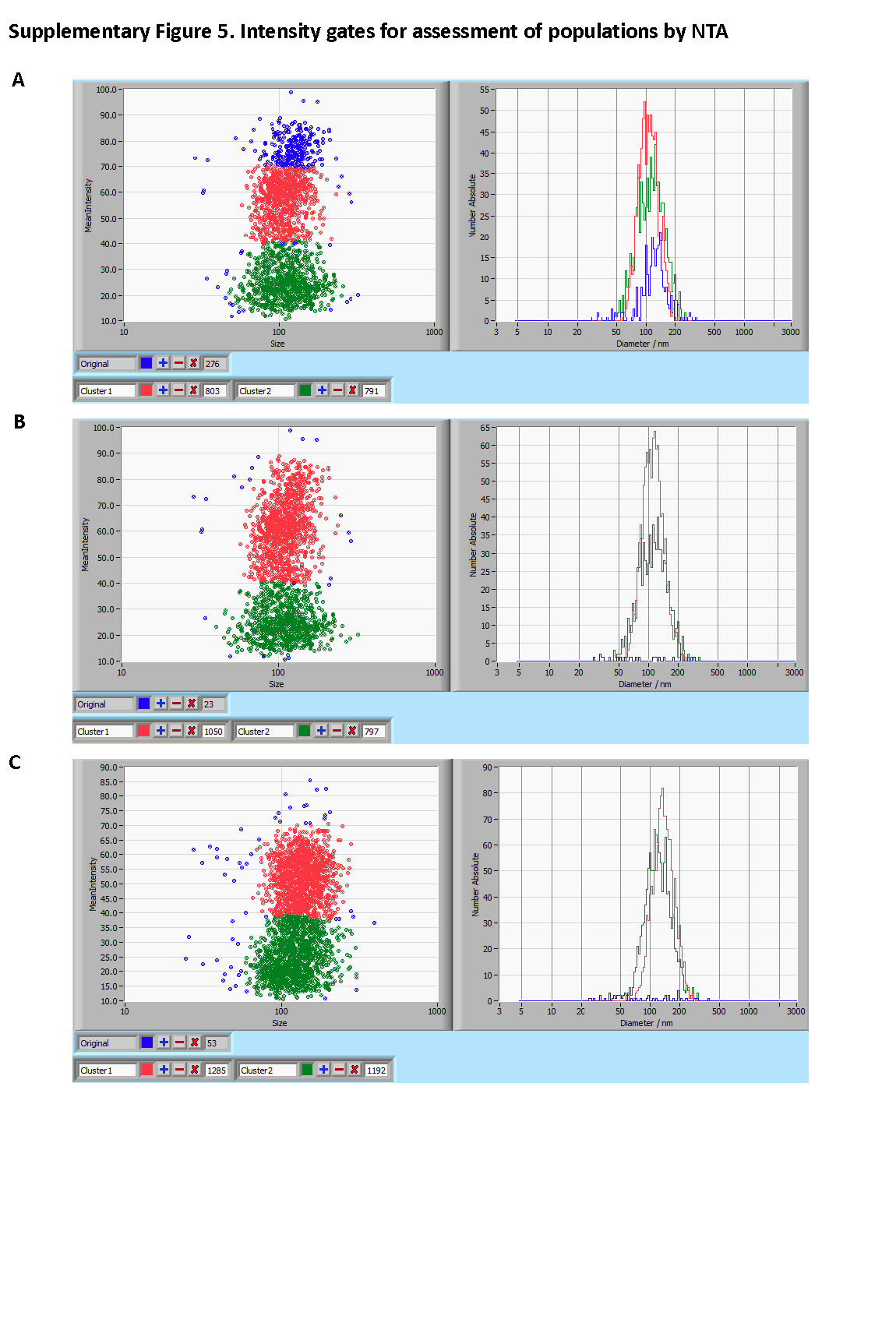

Supplement: Supplementary file 6 — Supplementary Figure 5: Intensity gates for assessment of populations by NTA. Data from NTA measurements of PS (A, B) and SS (C) were used to assign gates based on intensity. Note that (A) and (B) are the same data with different gates. Left panels are intensity vs. diameter plots. Right panels are abundance vs. diameter for each indicated (color‐coded) intensity gate. [file JEV2-10-e12079-s006.jpg]

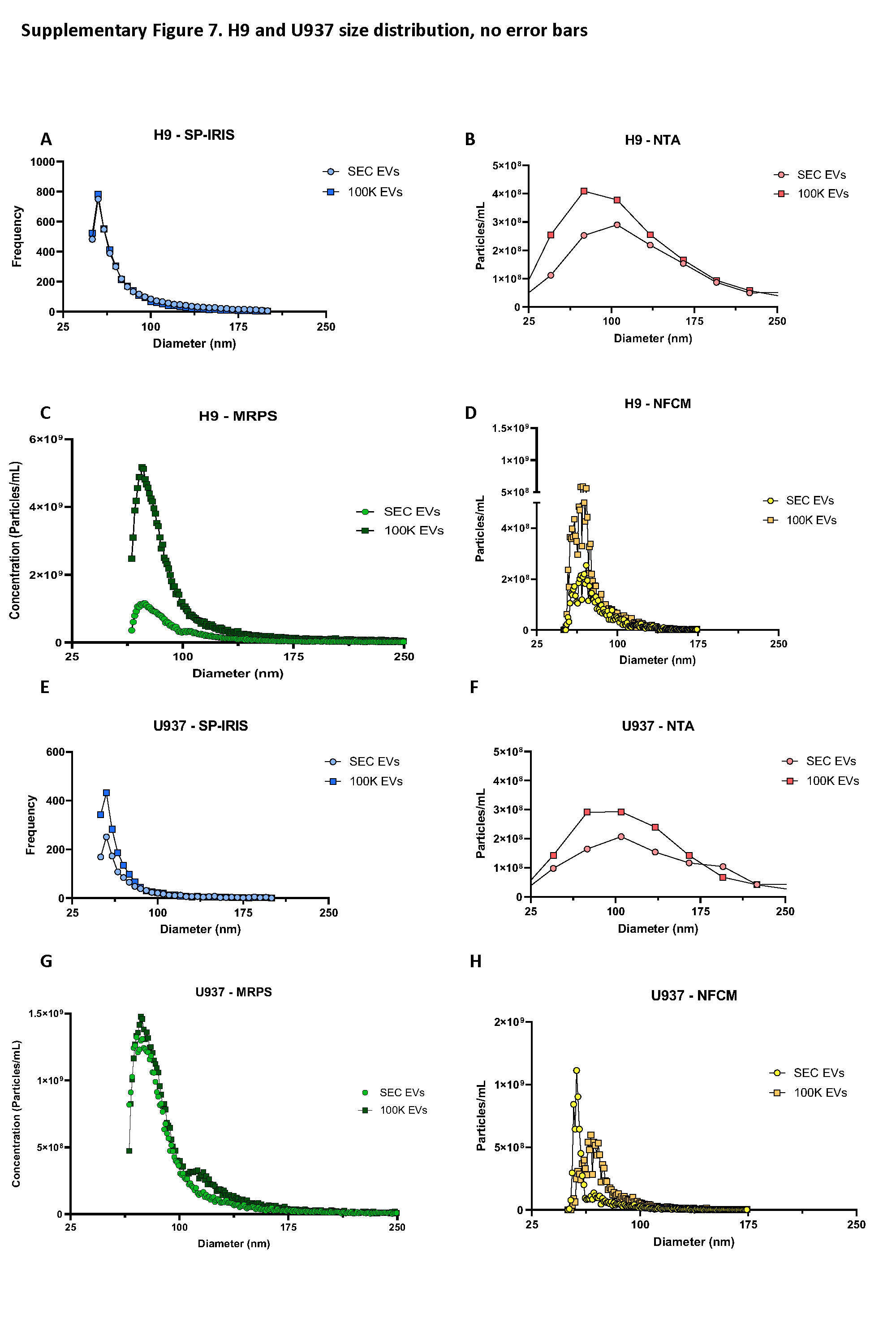

Supplement: Supplementary file 7 — Supplementary Figure 6: SP‐IRIS background fluorescence for SS and PS. SP‐IRIS fluorescence detection using fluorescently labelled anti‐CD81, anti‐CD63, and anti‐CD9 after drying (A) SS and (B) PS onto SP‐IRIS chips and measuring particles dried onto spots corresponding to the four antibody groups (n = 3 chips per group and 3 spots per antibody per chip; mean and SD are indicated by bars and whiskers). [file JEV2-10-e12079-s005.jpg]

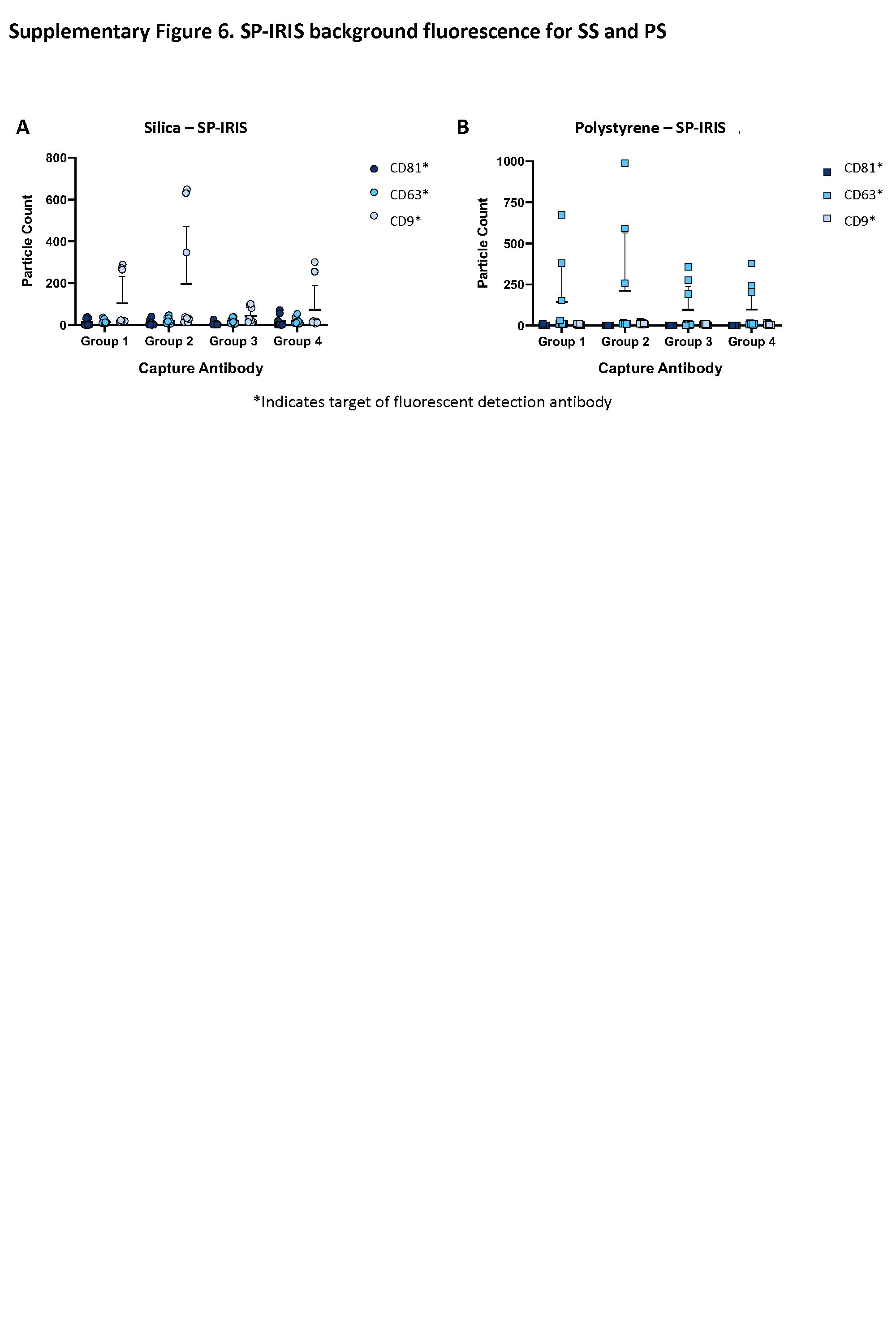

Supplement: Supplementary file 8 — Supplementary Figure 7: H9 and U937 EV size distribution, no error bars. This figure depicts the same data as shown in Figure 4, but without error bars for clarity. [file JEV2-10-e12079-s003.jpg]

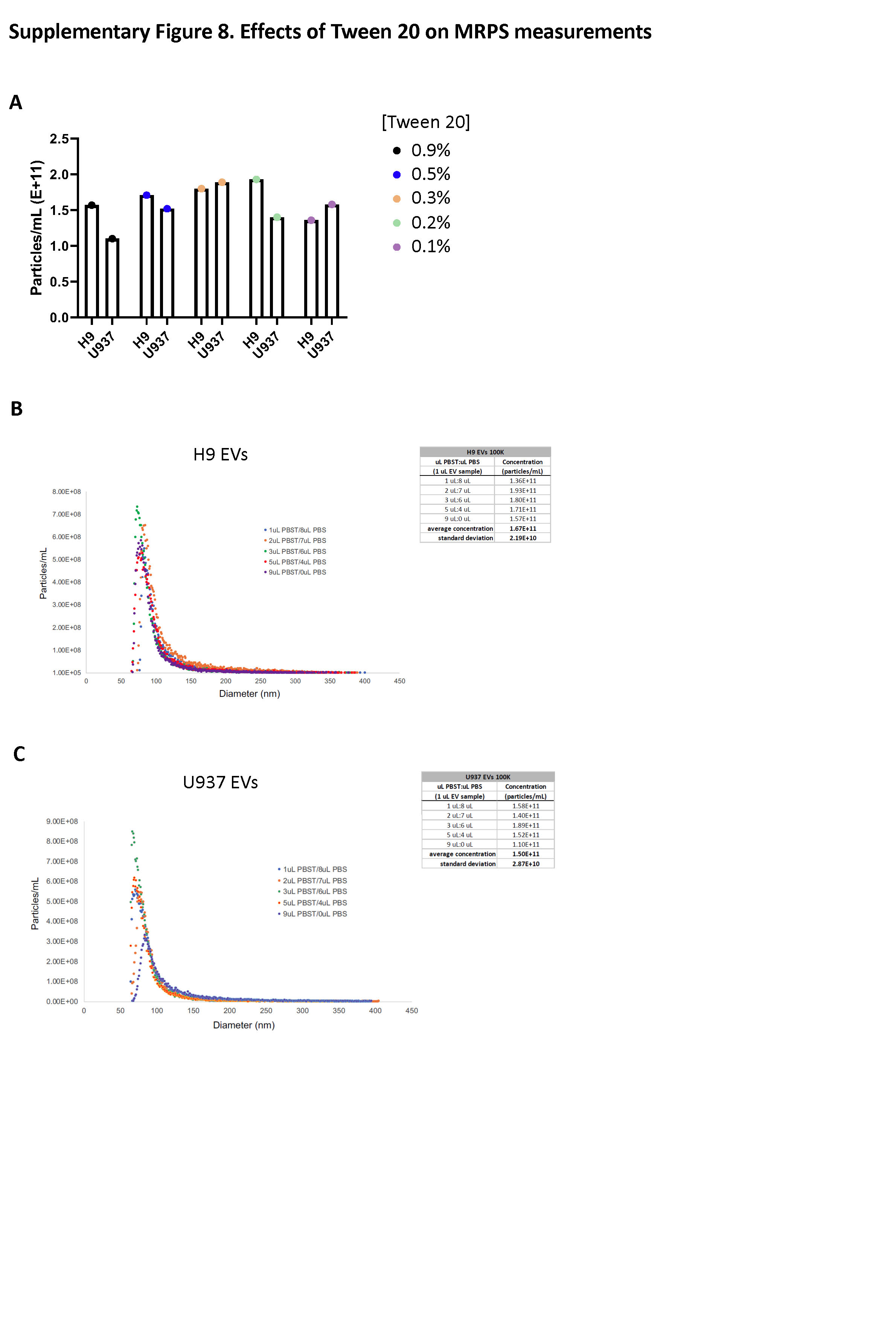

Supplement: Supplementary file 9 — Supplementary Figure 8: Effects of Tween 20 on MRPS measurements. H9 and U937‐derived EVs were mixed with Tween 20 to final concentrations ranging from 0.1% to 0.9% and measured by MRPS. (A) Particle counts. (B) and (C) depict size distributions for H9 and U937 EVs, respectively, along with insets displaying concentrations. [file JEV2-10-e12079-s010.jpg]
